# Objective and automatic assessment approach for diagnosing attention-deficit/hyperactivity disorder based on skeleton detection and classification analysis in outpatient videos

**DOI:** 10.1186/s13034-024-00749-5

**Published:** 2024-05-27

**Authors:** Chen-Sen Ouyang, Rei-Cheng Yang, Rong-Ching Wu, Ching-Tai Chiang, Yi-Hung Chiu, Lung-Chang Lin

**Affiliations:** 1 Department of Information Management, National Kaohsiung University of Science and Technology, No.1, University Rd., Yanchao District, Kaohsiung City, 824005 Taiwan; 2 Departments of Pediatrics, Kaohsiung Medical University Hospital, Kaohsiung Medical University, #100, Tzyou 1st Rd., Sanmin District, Kaohsiung City, 80756 Taiwan; 3 Department of Electrical Engineering, I-Shou University, No.1, Sec. 1, Syuecheng Rd., Dashu District, Kaohsiung City, 84001 Taiwan; 4 Department of Computer and Communication, National Pingtung University, No.4-18, Minsheng Rd., Pingtung City, 900391 Pingtung County Taiwan; 5 Department of Information Engineering, I-Shou University, No.1, Sec. 1, Syuecheng Rd., Dashu District, Kaohsiung City, 84001 Taiwan; 6 Department of Pediatrics, School of Medicine, College of Medicine, Kaohsiung Medical University, No.100, Shih-Chuan 1st Road, Sanmin District, Kaohsiung City, 807378 Taiwan

**Keywords:** ADHD, Machine learning, Skeleton detection, OpenPose, Variance analysis, Cutoff

## Abstract

**Background:**

Attention-deficit/hyperactivity disorder (ADHD) is diagnosed in accordance with *Diagnostic and Statistical Manual of Mental Disorders, Fifth Edition* criteria by using subjective observations and information provided by parents and teachers. However, subjective analysis often leads to overdiagnosis or underdiagnosis. There are two types of motor abnormalities in patients with ADHD. First, hyperactivity with fidgeting and restlessness is the major diagnostic criterium for ADHD. Second, developmental coordination disorder characterized by deficits in the acquisition and execution of coordinated motor skills is not the major criterium for ADHD. In this study, a machine learning-based approach was proposed to evaluate and classify 96 patients into ADHD (48 patients, 26 males and 22 females, with mean age: 7y6m) and non-ADHD (48 patients, 26 males and 22 females, with mean age: 7y8m) objectively and automatically by quantifying their movements and evaluating the restlessness scales.

**Methods:**

This approach is mainly based on movement quantization through analysis of variance in patients’ skeletons detected in outpatient videos. The patients’ skeleton sequence in the video was detected using OpenPose and then characterized using 11 values of feature descriptors. A classification analysis based on six machine learning classifiers was performed to evaluate and compare the discriminating power of different feature combinations.

**Results:**

The results revealed that compared with the non-ADHD group, the ADHD group had significantly larger means in all cases of single feature descriptors. The single feature descriptor “thigh angle”, with the values of 157.89 ± 32.81 and 15.37 ± 6.62 in ADHD and non-ADHD groups (*p* < 0.0001), achieved the best result (optimal cutoff, 42.39; accuracy, 91.03%; sensitivity, 90.25%; specificity, 91.86%; and AUC, 94.00%).

**Conclusions:**

The proposed approach can be used to evaluate and classify patients into ADHD and non-ADHD objectively and automatically and can assist physicians in diagnosing ADHD.

## Background

Attention-deficit/hyperactivity disorder (ADHD) is among the most common childhood behavioral disorders. A national survey conducted in 2016 revealed that 9.4% of children in the United States had been diagnosed as having ADHD and that 8.4% currently had ADHD [1, 2]. Currently, ADHD is diagnosed in accordance with *Diagnostic and Statistical Manual of Mental Disorders (DSM), Fifth Edition* (*DSM-V*) criteria [3]. In clinical practice, the ADHD diagnosis is often limited to subjective diagnosis of parents and teachers or that objective diagnosis is difficult and requires the input of an experienced clinician, results of standardized rating scales, and input from multiple informants across various settings [4]. There are two types of motor abnormalities in patients with ADHD, including hyperactivity and coordination impairment [5]. Hyperactivity with fidgeting and restlessness is the major diagnostic criterium for ADHD [6–8]. However, developmental coordination disorder characterized by deficits in the acquisition and execution of coordinated motor skills is not the major criterium for ADHD. In the present study, we tried to use OpenPose to quantify their movements and evaluate the restlessness scales in patients with ADHD. Several studies have objectively measured movement patterns in individuals with ADHD. However, these studies have numerous limitations. First, these studies have used accelerometers (actigraphy and inertial measurement units) that require the device to be attached to the participant’s body [9], limiting their ecological validity. Second, studies have employed infrared devices, which is easily interfered by light or other noise. In addition, infrared usually requires the use of special detection and software equipment [10]. Third, other studies have used impulse-radio ultra-wideband radar for monitoring hyperactive individuals with ADHD and healthy controls during a 22-min continuous performance test (CPT). Although this is a noncontact method, the surrounding moving objects of the CPT environment will interfere with radar detection and CPT is not a naturalistic setting [11]. In the present study, we used OpenPose to detect body movements in patients with ADHD by a regular camera. It is a convenient, time-saving, and noncontact method. In addition, we conduct detection during regular consultation and will not affect normal visiting behavior.

OpenPose, a posture-tracking algorithm that uses deep learning, was has become an essential tool for human posture tracking [12]. OpenPose is a real-time, multiperson system that can detect 135 facial, body, hand, and foot feature points simultaneously by a single image [12, 13]. Patients’ images and activities can be recorded when they are sitting in a consulting room only by a simple camera. OpenPose has been used to diagnose and monitor epilepsy [14], Parkinson’s disease [15], and osteoarthritis (OA) [16] as well as track multiperson movements on a single image [12]. A study used OpenPose to track the movements of patients with epilepsy. Their findings indicated that this method provided improved posture-tracking information in clinical settings. The accuracy rates in head pose estimation in all patients were over 97% [17]. In patients with Parkinson’s disease, Sato et al. used OpenPose to analyze daily clinical movies recorded from the frontal view and determine continuous gait features from these movies by extracting body joint coordinates with OpenPose. Their results demonstrated a parkinsonian gait with obvious freezing gait and involuntary oscillations. The periodicity of each gait sequence can be calculated by an autocorrelation function–based statistical distance metric. Participants’ baseline disease status was significantly correlated with the metric [15, 18]. OpenPose was used to replace an expensive gait analysis tool applied for detecting the knee adduction moment (KAM) in patients with knee OA by Boswell et al. The KAM was compared between 64 participants with and without OA with natural and modified walking (foot progression angle modifications) through two-dimensional video analysis. The results demonstrated that on the basis of the positions of anatomical landmarks determined through motion tracking, a neural network accurately predicted the peak KAM during natural and modified walking. The results also validated the feasibility of measuring the peak KAM on the basis of positions determined using OpenPose [16]. To accurately and objectively classify the patients with and without ADHD in a consulting room, we evaluated movements by using the OpenPose system and then analyzed the movements of patients with and without ADHD.

## Methods

### Overview

**Fig. 1:**
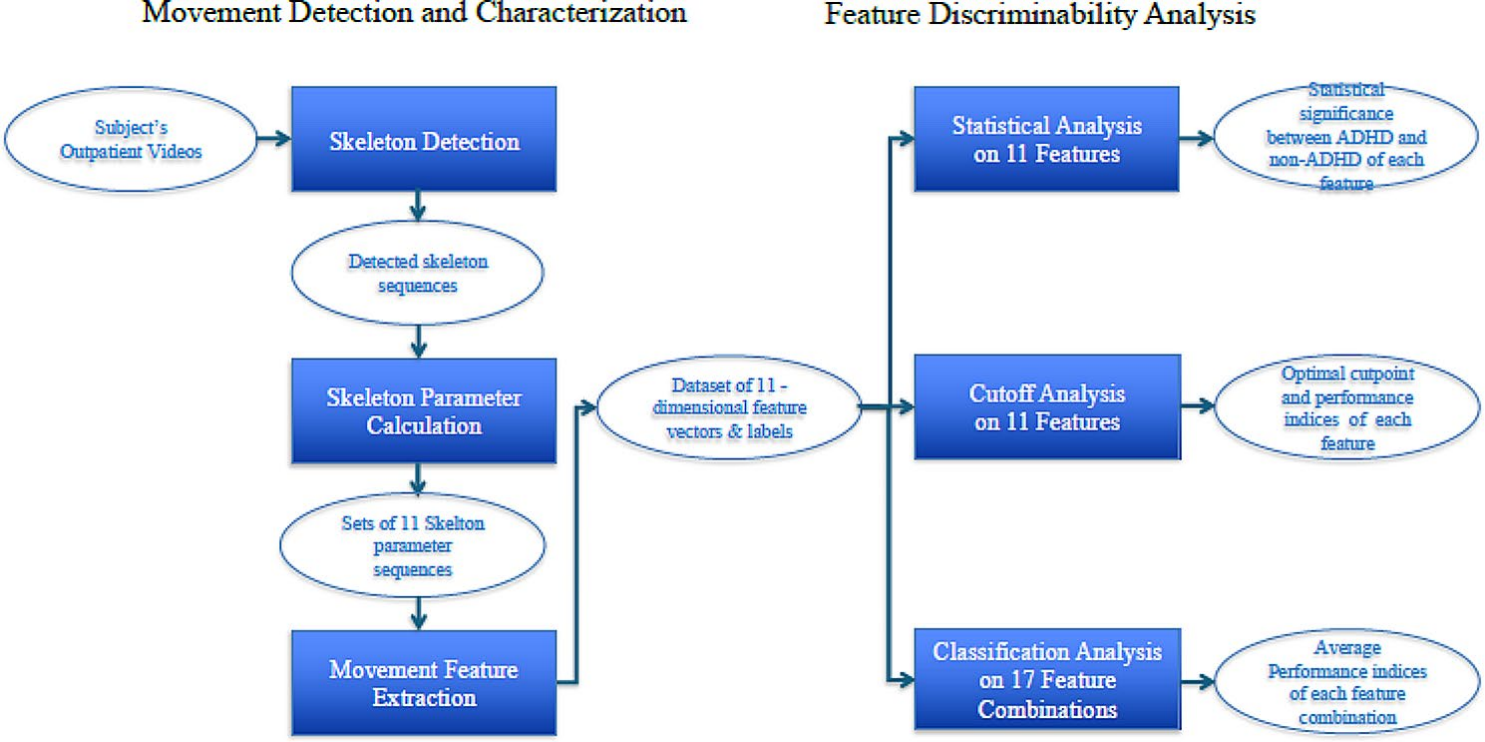
Flowchart of the proposed approach

Our method included two phases, i.e., movement detection and characterization and feature discriminability analysis, as shown in Fig. 1. In the phase of movement detection and characterization, skeleton detection was performed by the “openpose” on each subject’s outpatient video to detect the corresponding skeleton sequence. Then, the corresponding set of 11 skeleton parameter sequences was calculated from each subject’s detected skeleton sequence. After that, the average variance of each of 11 skeleton parameter sequences was calculated by a sliding window approach, resulting in an 11-dimensional feature vector. Finally, the dataset of all subjects’ feature vectors and corresponding labels was obtained. In the next phases, i.e., feature discriminability analysis, the statistical comparison, cutoff, and classification were performed on the obtained dataset to verify the discriminability of each feature and each feature combination. For each feature, the statistical comparison analysis was applied to present the statistical significance between ADHD and non-ADHD; the cutoff analysis was used to find the optimal cutpoint and calculate the corresponding performance indices. To further discover the discriminability of multiple features, the classification analysis based on 17 feature combinations and six well-known machine learning classifiers was performed, and the corresponding performance indices and ranking were calculated.

### Participants

We included 48 children (26 males and 22 females, mean age: 7 years 6 months ± 2 years 2 month) with ADHD (ADHD group) and 48 children (26 males and 22 females, mean age: 7 years 8 month ± 2 years 2 months) without ADHD (non-ADHD group), all of whom were examined by a pediatric neurologist and asked to sit on a chair for data recording. A diagnosis of ADHD was made in accordance with *DSM-V* criteria. ADHD severity was evaluated using the 26-item Swanson, Nolan, and Pelham Rating Scale (SNAP-IV), including 18 items on ADHD symptoms (nine related to inattentiveness and nine related to hyperactivity/impulsiveness) and eight items on oppositional defiant disorder symptoms specified in *DSM, Fourth Edition* criteria. Each item measures the frequency of the appearance of symptoms or behaviors, in which the observer indicates whether the behavior occurs “not at all”, “just a little”, “quite a bit”, or “very much”. The items were scored by observer on a 4-point scale from 0 (not at all) to 3 (very much). The ADHD is divided into three major type: inattentiveness (ADHD-I, children with this type of ADHD exhibit no or few signs of hyperactivity or impulsivity. Instead, the children will get distracted easily and difficult to pay attention), hyperactivity/impulsivity (ADHD-H, the children will demonstrate signs of hyperactivity and the need to move constantly and display impulsive behavior. They show no or few signs of getting distracted or inattention), and combined (ADHD-C, the children will demonstrate impulsive and hyperactive behavior and get distracted easily). To prevent biased comparison, children with a history of intellectual disability, drug abuse, head injury, or psychotic disorders were excluded from the ADHD group. The diagnoses in the patients without ADHD were headache, epilepsy, and dizziness, which are common in pediatric neurology. Written informed consent was obtained by a participant’s family member or legal guardian after the procedure had been explained. In addition, informed consent was also obtained from them for the publication of their children’s images. This study was approved by the Institutional Review Board of Kaohsiung Medical University Hospital (KMUIRB-SV(I)- 20190060).

### Movement detection and characterization

**Fig. 2:**
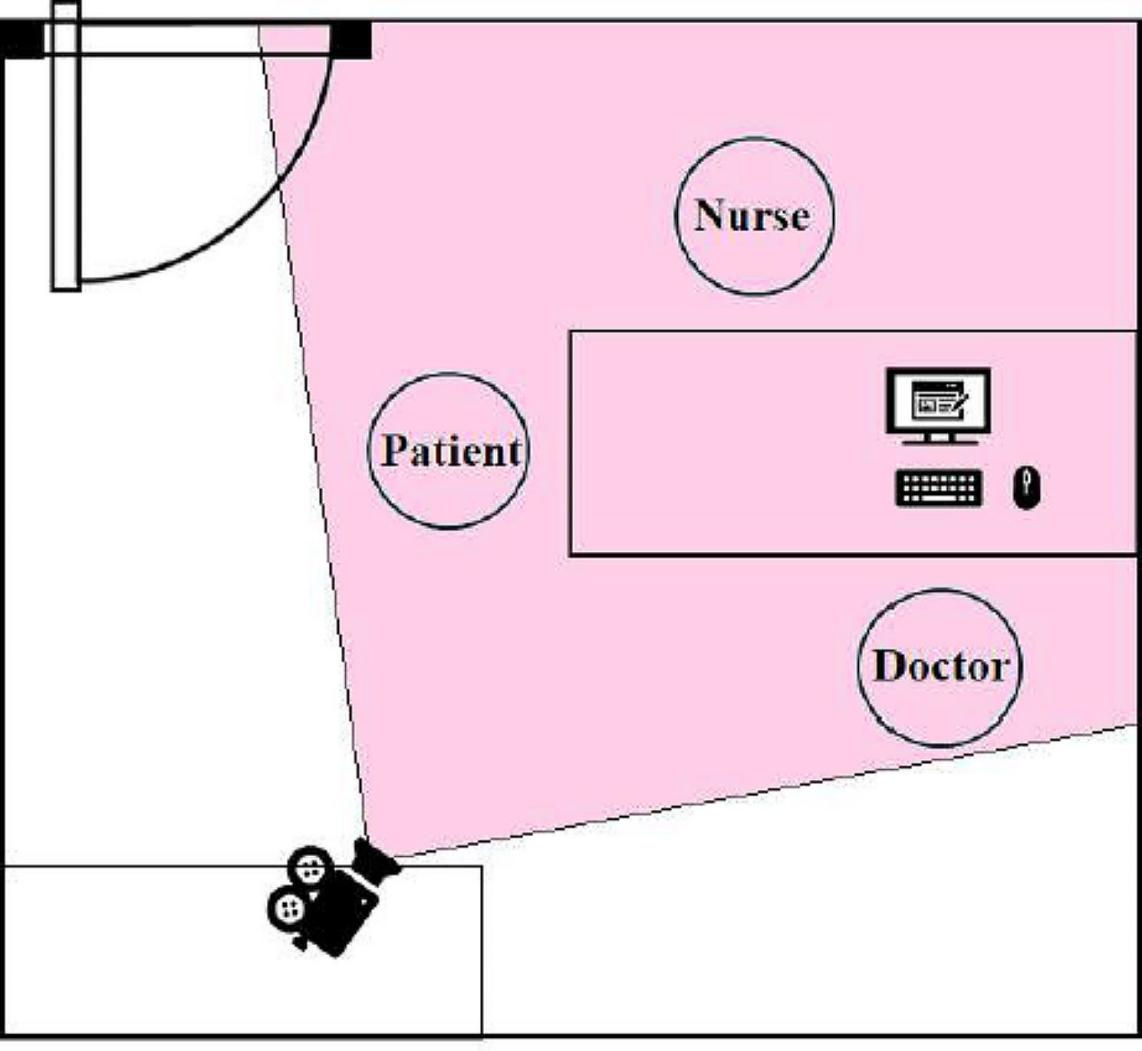
The camera’s position and view in the consultation room

**Fig. 3:**
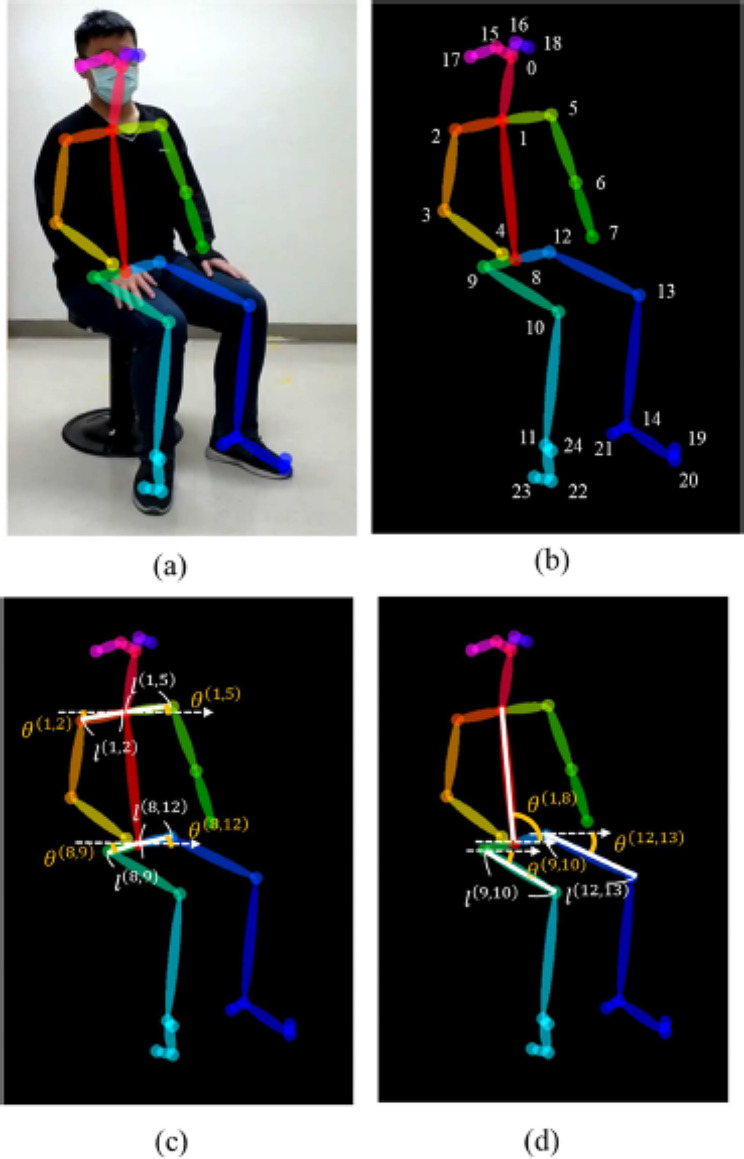
Example of a patient’s skeleton detection. A detected patient’s skeleton represented by 25 key points and the corresponding skeleton parameters: **a** detected skeletons; **b** 25 key points; **c** shoulder-related and hip-related parameters; and d) thigh-related and trunk-related parameters

We propose an objective and automatic approach to evaluate the movements of patients with ADHD and compare them with those of patients without ADHD. This approach is mainly based on movement quantization through the analysis of variance in patients’ skeletons detected automatically in outpatient videos (specifically, 4–6-min video recordings per patient). The 2D camera (I-Family IF-005D) was used to capture movement videos of each patient, with video recordings obtained at a frame rate of 30 Hz for each patient and a resolution of 1280 × 720. The camera was placed in a fixed position in the consulting room, as shown in Fig. 2. To minimize comparison bias, only the initial 4-min video recording was considered for analysis. To quantify the patients’ movements in an outpatient video objectively and automatically, we used OpenPose for detecting the patient’s skeleton in each video frame. This study employed two-dimensional (2D) real-time multiperson skeleton detection [12]. Figure 3 presents an example of the detected skeleton of a patient represented by 25 key points (joints): nose (0), neck (1), right shoulder (2), right elbow (3), right wrist (4), left shoulder (5), left elbow (6), left wrist (7), middle hip (8), right hip (9), right knee (10), right ankle (11), left hip (12), left knee (13), left ankle (14), right eye (15), left eye (16), right ear (17), left ear (18), left big toe (19), left small toe (20), left heel (21), right big toe (22), right small toe (23), and right heel (24). The detection result of each skeleton was represented by the 2D coordinates of these 25 joints in the image domain.

Assume $$ P^{t} = \{ \overset{\lower0.5em\hbox{$\smash{\scriptscriptstyle\rightharpoonup}$}} {p} _{i} ^{t}|i = {{0,2}}, \ldots,24\} $$ is the set of the 25 detected joints in the $$t$$th frame of an outpatient video. Let the frame coordinate of the $$i$$th joint $${{ \overset{\lower0.5em\hbox{$\smash{\scriptscriptstyle\rightharpoonup}$}}{{p}}}_{i}}^{t}$$ be represented by $$({{x}_{i}}^{t},{{y}_{i}}^{t})$$, where $${{x}_{i}}^{t}\in \{{0,1},\dots,{N}_{x}-1\}$$ and $${{y}_{i}}^{t}\in \{{0,1},\dots,{N}_{y}-1\}$$. $${N}_{x}$$ and $${N}_{y}$$ are the frame’s width and height, respectively. On the basis of the natural connections (bones) between some pairs of joints, several bone vectors were defined, such as the right shoulder $${{ \overset{\lower0.5em\hbox{$\smash{\scriptscriptstyle\rightharpoonup}$}}{{b}} }_{1,2}}^{t}=({{x}_{2}}^{t}-{{x}_{1}}^{t},{{y}_{2}}^{t}-{{y}_{1}}^{t})$$ from the neck joint $$ \overset{\lower0.5em\hbox{$\smash{\scriptscriptstyle\rightharpoonup}$}}{{p}}_{{1}}^{t}$$ to the right shoulder $${{ \overset{\lower0.5em\hbox{$\smash{\scriptscriptstyle\rightharpoonup}$}}{{p}} }_{2}}^{t}$$ and the left shoulder $${{ \overset{\lower0.5em\hbox{$\smash{\scriptscriptstyle\rightharpoonup}$}}{{b}} }_{{1,5}}}^{t}=({{x}_{5}}^{t}-{{x}_{1}}^{t},{{y}_{5}}^{t}-{{y}_{1}}^{t})$$ from the neck joint $${{ \overset{\lower0.5em\hbox{$\smash{\scriptscriptstyle\rightharpoonup}$}}{{p}} }_{1}}^{t}$$ to the left shoulder $${{ \overset{\lower0.5em\hbox{$\smash{\scriptscriptstyle\rightharpoonup}$}}{{p}} }_{5}}^{t}$$. To extract the skeleton’s features for characterizing patients’ movements and differentiate them between the ADHD and non-ADHD groups in outpatient videos, two types of skeleton parameters were defined, namely bone length and bone angle. For a bone vector $${{ \overset{\lower0.5em\hbox{$\smash{\scriptscriptstyle\rightharpoonup}$}}{{b}} }_{i,j}}^{t}=({{x}_{j}}^{t}-{{x}_{i}}^{t},{{y}_{j}}^{t}-{{y}_{i}}^{t})$$, bone length $${l}_{i,j}^{t}$$ was defined as follows:

1 $${l}_{i,j}^{t}=\sqrt[2]{{\left({{x}_{j}}^{t}-{{x}_{i}}^{t}\right)}^{2}+{\left({{y}_{j}}^{t}-{{y}_{i}}^{t}\right)}^{2}},$$

Bone angle ${\theta }_{i,j}^{t}$ was defined as follows:

2 $${\theta }_{i,j}^{t}=\left|{\text{tan}}^{-1}\left[\frac{\left({{y}_{j}}^{t}-{{y}_{i}}^{t}\right)}{\left({{x}_{j}}^{t}-{{x}_{i}}^{t}\right)}\right]\times \frac{180}{\pi }\right|.$$

On the basis of the patients’ movements observed in outpatient videos, six bone vectors, namely the right shoulder, left shoulder, right hip, left hip, right thigh, and trunk, were selected, and the corresponding lengths and angles were calculated. In addition to the right shoulder and left shoulder defined previously, four bone vectors were defined as follows:

1. Right hip $${{ \overset{\lower0.5em\hbox{$\smash{\scriptscriptstyle\rightharpoonup}$}}{{b}} }_{{8,9}}}^{t}=({{x}_{9}}^{t}-{{x}_{8}}^{t},{{y}_{9}}^{t}-{{y}_{8}}^{t})$$ from the middle hip joint $${{\overset{\lower0.5em\hbox{$\smash{\scriptscriptstyle\rightharpoonup}$}}{p}}_{8}}^{t}$$ to the right hip $${{\overset{\lower0.5em\hbox{$\smash{\scriptscriptstyle\rightharpoonup}$}}{p}}_{9}}^{t}$$;
2. Left hip $${{ \overset{\lower0.5em\hbox{$\smash{\scriptscriptstyle\rightharpoonup}$}}{{b}} }_{{8,12}}}^{t}=({{x}_{12}}^{t}-{{x}_{8}}^{t},{{y}_{12}}^{t}-{{y}_{8}}^{t})$$ from the middle hip joint $${{\overset{\lower0.5em\hbox{$\smash{\scriptscriptstyle\rightharpoonup}$}}{p}}_{8}}^{t}$$ to the left hip $${{\overset{\lower0.5em\hbox{$\smash{\scriptscriptstyle\rightharpoonup}$}}{p}}_{12}}^{t}$$;
3. Right thigh $${{ \overset{\lower0.5em\hbox{$\smash{\scriptscriptstyle\rightharpoonup}$}}{{b}} }_{{9,10}}}^{t}=({{x}_{10}}^{t}-{{x}_{9}}^{t},{{y}_{10}}^{t}-{{y}_{9}}^{t})$$ from the right hip joint $${{\overset{\lower0.5em\hbox{$\smash{\scriptscriptstyle\rightharpoonup}$}}{p}}_{9}}^{t}$$ to the right knee joint $${{\overset{\lower0.5em\hbox{$\smash{\scriptscriptstyle\rightharpoonup}$}}{p}}_{10}}^{t}$$;
4. Trunk $${{ \overset{\lower0.5em\hbox{$\smash{\scriptscriptstyle\rightharpoonup}$}}{{b}} }_{{1,8}}}^{t}=({{x}_{8}}^{t}-{{x}_{1}}^{t},{{y}_{8}}^{t}-{{y}_{1}}^{t})$$ from the neck joint $${{\overset{\lower0.5em\hbox{$\smash{\scriptscriptstyle\rightharpoonup}$}}{p}}_{1}}^{t}$$ to the middle hip joint $${{\overset{\lower0.5em\hbox{$\smash{\scriptscriptstyle\rightharpoonup}$}}{p}}_{8}}^{t}$$.

The right thigh was selected instead of the left thigh because the left thigh was usually partially occluded by the right thigh owing to the seated position of the patient. The corresponding lengths and angles of all bone vectors except the trunk were calculated using Eqs. (1) and (2), respectively, resulting in five length-related skeleton parameters, namely ${l}_{1,2}^{t}$, ${l}_{{1,5}}^{t}$, ${l}_{{8,9}}^{t}$, ${l}_{{8,12}}^{t}$, and ${l}_{{9,10}}^{t}$, and five angle-related skeleton parameters, namely ${\theta }_{1,2}^{t}$, ${\theta }_{{1,5}}^{t}$, ${\theta }_{{8,9}}^{t}$, ${\theta }_{{8,12}}^{t}$, and ${\theta }_{{9,10}}^{t}$. The corresponding angle of trunk bone vector ${\theta }_{{8,1}}^{t}$ was calculated using the following equation:

3 $${\theta }_{{8,1}}^{t}=\left\{\begin{array}{ll}{\varphi }_{i,j}^{t},& if\,\, {\varphi }_{i,j}^{t}\ge 0\\ {\varphi }_{i,j}^{t}+180,& if \,\,{\varphi }_{i,j}^{t}<0\end{array}\right. {\text{where}} \,\,{\varphi }_{i,j}^{t}={\text{tan}}^{-1}\left[\frac{\left({{y}_{j}}^{t}-{{y}_{i}}^{t}\right)}{\left({{x}_{j}}^{t}-{{x}_{i}}^{t}\right)}\right]\times \frac{180}{\pi }.$$

Eleven skeleton parameters were extracted to characterize the detected skeleton in each frame of an outpatient video. For an outpatient video composed of $T$ frames, $T$ detected skeletons were present. The corresponding $T$ values of each skeleton parameter constituted a time series. Thus, 11 time series corresponding to 11 skeleton parameters were obtained to characterize the detected skeleton sequence in the video.

Let $${\varvec{l}}_{i,j}^{}=({l}_{i,j}^{1},{l}_{i,j}^{2},\dots,{l}_{i,j}^{T})$$ and $${\varvec{\theta }}_{i,j}^{}=({\theta }_{i,j}^{1},{\theta }_{i,j}^{2},\dots,{\theta }_{i,j}^{T})$$ be the two series of the length and angle, respectively, corresponding to bone vector $${{\overset{\lower0.5em\hbox{$\smash{\scriptscriptstyle\rightharpoonup}$}}{b}}_{i,j}}^{t}$$. To characterize the variation in values in each series, the averaged variances of series $${\varvec{l}}_{i,j}^{}$$ and $${\varvec{\theta }}_{i,j}^{}$$ were calculated using a sliding window approach:

4 $${\sigma }^{2}\left({\varvec{l}}_{i,j}^{}\right)=\frac{1}{K}\sum _{k=1}^{K}{\sigma }^{2}\left(\varvec{ \tilde{l}}_{i,j}^{k}\right), {\sigma }^{2}\left(\varvec{ \tilde{l}}_{i,j}^{k}\right)=\frac{1}{R-1}\sum _{t=r}^{r+R-1}{\left({l}_{i,j}^{t}-m\left({\stackrel{\sim}{\varvec{l}}}_{i,j}^{k}\right)\right)}^{2}$$

5 $${\sigma }^{2}\left({\varvec{\theta }}_{i,j}^{}\right)=\frac{1}{K}\sum _{k=1}^{K}{\sigma }^{2}\left(\varvec{ \tilde{\theta}}_{i,j}^{k}\right), {\sigma }^{2}\left(\varvec{ \tilde{\theta}}_{i,j}^{k}\right)=\frac{1}{R-1}\sum _{t=r}^{r+R-1}{\left({\theta }_{i,j}^{t}-m\left(\varvec{ \tilde{\theta}}_{i,j}^{k}\right)\right)}^{2}$$

where $${\varvec{ \tilde{\theta}}}_{i,j}^{k}=\left({l}_{i,j}^{r},{l}_{i,j}^{r+1},\dots, {l}_{i,j}^{r+R-1}\right)$$ and $${\varvec{ \tilde{\theta}}}_{i,j}^{k}=\left({\theta }_{i,j}^{r},{\theta }_{i,j}^{r+1}, \right.$$$$\left.\dots,{\theta }_{i,j}^{r+R-1}\right)$$, $$r=\left(k-1\right)\times R+1$$, are the $$k$$th subsequences of $${\varvec{l}}_{i,j}^{}$$ and $${\varvec{\theta }}_{i,j}^{}$$ with a window size of $$R$$; $$m\big({\varvec{ \tilde{l}}}_{i,j}^{k}\big)$$ and $$m\big({\varvec{ \tilde{\theta}}}_{i,j}^{k}\big)$$ are the corresponding means; $${\sigma }^{2}\big({\varvec{ \tilde{l}}}_{i,j}^{k}\big)$$ and $${\sigma }^{2}\big({\varvec{ \tilde{\theta}}}_{i,j}^{k}\big)$$ are the corresponding variances; and $$K$$ is the number of subsequences. Thus, 11 values of feature descriptors, $${\sigma }^{2}\left({\varvec{l}}_{1,2}^{}\right),$$ $${\sigma }^{2}\left({\varvec{l}}_{{1,5}}^{}\right),$$ $${\sigma }^{2}\left({\varvec{l}}_{{8,9}}^{}\right),$$ $${\sigma }^{2}\left({\varvec{l}}_{{8,12}}^{}\right),$$ $${\sigma }^{2}\left({\varvec{l}}_{{9,10}}^{}\right),$$ $${\sigma }^{2}\left({\varvec{\theta }}_{1,2}^{}\right),$$ $${\sigma }^{2}\left({\varvec{\theta }}_{{1,5}}^{}\right),$$ $${\sigma }^{2}\left({\varvec{\theta }}_{{8,9}}^{}\right),$$ $${\sigma }^{2}\left({\varvec{\theta }}_{{8,12}}^{}\right),$$ $${\sigma }^{2}\left({\varvec{\theta }}_{{9,10}}^{}\right),$$ and $${\sigma }^{2}\left({\varvec{\theta }}_{{1,8}}^{}\right)$$, were obtained to characterize the patient’s movement in an outpatient video. Finally, a two-dimensional dataset matrix with $$96$$ rows and 12 columns was obtained for the following feature discriminability analysis. Note that each row corresponds to one subject’s 11 feature descriptor values (i.e., 11 averaged variances of skeleton parameters’ series detected from the initial 4-minute video recording) and one class label (ADHD or non-ADHD).

### Feature discriminability analysis

To evaluate and compare the discriminating power of different features between the ADHD and non-ADHD groups, we determined an optimal cutoff. We adopted bootstrapping to prevent highly variable results and systematic overestimation of the out-of-sample performance. Let $S=\left\{\right({f}_{n},{c}_{n}\left)\right|n=1,2,\dots,96\}$ be the original sample set of the feature descriptor to be evaluated, where ${f}_{n}$ and ${c}_{n}$ are the corresponding value and class label, respectively, of the *n*th patient. Each time, a so-called “bootstrap” or in-bag sample set $\tilde{S}$, with the same size (i.e., 96) as that of $S$, was drawn randomly with replacement, and samples not drawn constituted a so-called “out-of-bag sample set.” On average, an in-bag sample set $\tilde{S}$ included 63.2% of all the samples of original sample set $S$ because some samples were drawn multiple times [19]. An optimal cutpoint was determined by computing the performance index of discriminative ability at each value of the feature descriptor in the in-bag sample set $\tilde{S}$, and then selecting the feature value with the largest Youden index (defined as $sensitivity+ specificity-1$) value as the optimal cutpoint. Note that $Sensitivity$ was the percentage of the correct prediction of the class “ADHD” for all patients in the ADHD group, while $Specificity$ was the percentage of the correct prediction of the class “non-ADHD” for all patients in the non-ADHD group. After that, the obtained optimal cutpoint was applied to the out-of-bag sample set, and the corresponding four performance indices, namely $accuracy$, $sensitivity$, $specificity$, and area under the receiver operating characteristic curve $(AUC)$, were calculated. $Accuracy$ was the percentage of the correct prediction of the “ADHD” or “non-ADHD” class for all patients in both the groups. $AUC$ was plotted with pairs of values of $1- specificity$ and $sensitivity$ corresponding to binary classification results obtained using different classification threshold values. The above process of optimal cutpoint searching in an in-bag sample set and testing in the corresponding out-of-bag sample set was repeated 100 times and the 100 different optimal cutoff values each with the corresponding values of four test performance indices were obtained. Finally, the average optimal cutpoint and four average test performance indices were calculated for evaluating the feature descriptor’s discriminating power between the ADHD and non-ADHD groups based on the cutoff analysis.

To evaluate the discriminating power of different feature combinations between the ADHD and non-ADHD groups, we performed classification analysis based on six machine learning classifiers and employed hyperparameter tuning with five-fold cross-validation to identify the most suitable model parameters. The adaptive boosting (AdaBoost) model’s weak classifiers were implemented with the classification and regression tree (CART) algorithm, and the corresponding parameter n-estimators were optimized within {1, 5, 10, 20, 30, 50}. The decision tree classifiers were implemented with CART algorithm, and the corresponding parameter max-depth was optimized within {1, 2, 3, 5, 7}. The k-nearest neighbors (KNN) model’s parameter n-neighbors was optimized within {1, 2, 3}. The random forest model’s parameters max-features, max-depth, and n-estimators were optimized with a grid search within {1, 2, 3}, {1, 2, 3, 5, 7}, and {1, 5, 10, 20, 30, 50}, respectively. The support vector machine (SVM) model’s kernel type was set as the radial basis function, and the corresponding parameters gamma and C were optimized with a grid search within {50, 100, 300, 500} and {0.001, 0.01, 0.1, 1}, respectively. The extreme gradient boosting (XGBoost) model’s weak classifiers were implemented with the CART algorithm, and the corresponding parameters learning rate, max-depth and n-estimators were optimized with a grid search within {0.1, 0.2, 0.3}, {1, 2, 3, 5, 7}, and {1, 5, 10, 20, 30, 50}, respectively. Seventeen feature combinations were evaluated and compared, including the 11 single features and six additional feature combinations—two thigh-related features (thigh-related) $$\{{\sigma }^{2}\left({\varvec{l}}_{{9,10}}^{}\right),{\sigma }^{2}\left({\varvec{\theta }}_{{9,10}}^{}\right)\},$$ four shoulder-related features (shoulder-related) $$\{{\sigma }^{2}\left({\varvec{l}}_{1,2}^{}\right), {\sigma }^{2}\left({\varvec{l}}_{{1,5}}^{}\right), {\sigma }^{2}\left({\varvec{\theta }}_{1,2}^{}\right),{\sigma }^{2}\left({\varvec{\theta }}_{{1,5}}^{}\right)\},$$ four hip-related features (hip-related) $$\{{\sigma }^{2}\left({\varvec{l}}_{{8,9}}^{}\right),{\sigma }^{2}\left({\varvec{l}}_{{8,12}}^{}\right), $$$$\{{\sigma }^{2}\left({\varvec{\theta }}_{{8,9}}^{}\right), {\sigma }^{2}\left({\varvec{\theta }}_{{8,12}}^{}\right)\},$$ five length-related features (length-related) $$\{ {\sigma }^{2}\left({\varvec{l}}_{1,2}^{}\right), {\sigma }^{2}\left({\varvec{l}}_{{1,5}}^{}\right), {\sigma }^{2}\left({\varvec{l}}_{{8,9}}^{}\right),$$$${\sigma }^{2}\left({\varvec{l}}_{{8,12}}^{}\right)\},{\sigma }^{2}\left({\varvec{l}}_{{9,10}}^{}\right),$$ six angle-related features (angle-related)$$\{{\sigma }^{2}\left({\varvec{\theta }}_{{9,10}}^{}\right), {\sigma }^{2}\left({\varvec{\theta }}_{1,2}^{}\right), {\sigma }^{2}\left({\varvec{\theta }}_{{1,5}}^{}\right), {\sigma }^{2}\left({\varvec{\theta }}_{{8,9}}^{}\right), $$$$ {\sigma }^{2}\left({\varvec{\theta }}_{{8,12}}^{}\right),$$ $${\sigma }^{2}\left({\varvec{\theta }}_{{8,1}}^{}\right)\},$$ and all 11 features (all).

For each feature combination, the corresponding dataset comprised 48 feature vectors with “ADHD” labels and 48 with “non-ADHD” labels. To minimize the bias of model evaluation, the resampling strategy of 10-fold cross-validation was repeated 10 times. In each repetition, the dataset was equally and randomly partitioned into 10 folds, with each being composed of four to five “ADHD” and four to five “non-ADHD” feature vectors. Next, a fold was selected as the test dataset, and the remaining folds were selected as the training dataset. This training–test partitioning process was repeated 10 times, with each of the 10 folds being used only once as the test dataset. Moreover, the resampling strategies of 8:2 and 6:4 training-test random splits (holdout methods) with 100 repeats were also be applied for comparison. A total of 100 pairs of training and test datasets were obtained in each resampling strategy. For each pair, the training dataset was used to train the considered classifier and the test dataset was used to evaluate the trained classifier’s classification performance on the basis of four classification performance indices, namely accuracy, sensitivity, specificity, and AUC. The 100 values of each index corresponding to the 100 test datasets were averaged to estimate the classification test performance of the classifier. The larger the values of all four indices were, the stronger the discriminating power of the combination of the feature set and classifier was. To compare the discriminating power of the 17 feature sets across the six classifiers, the averaged ranking of each feature set corresponding to each classification performance index was calculated by averaging the feature set’s ranks in the corresponding index’s results of six classifiers. The smaller the averaged rank values of all four indices were, the stronger the discriminating power of the feature set was.

### Statistical analysis

All statistical analyses were conducted using SAS (v9.3; SAS Institute, Cary, NC, USA). Data are presented as means ± standard deviation. Measurements between patients with and without ADHD were conducted using the two-sample *t* test. *P* < 0.05 was considered statistically significant.

## Results

We enrolled 48 patients with ADHD and 48 age- and sex-matched patients without ADHD (Table 1). There was no significant difference in age between with and without ADHD (*p* = 0.647). Each group comprised 26 boys and 22 girls. Twenty boys had ADHD-C, four boys had ADHD-I, and two boys had ADHD-H; 16 girls had ADHD-C; and six girls had ADHD-I. Among ADHD subtypes, ADHD-C and ADHD-H are the most prevalent (78.0–81.7%), followed by ADHD-I (18.3–22.0%) in the literatures [20–22]. In this study, 38 of the 48 patients had ADHD-C or ADHD-H. Therefore, most of the recruited patients exhibited hyperactive symptoms. The SNAP-IV scores obtained from parents and teachers were 36.88 ± 16.05 and 34.09 ± 16.19, respectively.

**Table 1:** Demographic data of patients with ADHD

Sex (M/F)	Age	Index	Parent’s SNAP score	Teacher’s SNAP score
26/22	7y6m ± 2y2m	Inattention	13.62 ± 5.40	15.11 ± 6.49
		Hyperactivity-impulsivity	12.78 ± 7.28	11.58 ± 7.54
		Oppositional	10.39 ± 6.78	7.39 ± 6.47
		Total	36.88 ± 16.05	34.09 ± 16.19

**Fig. 4:**
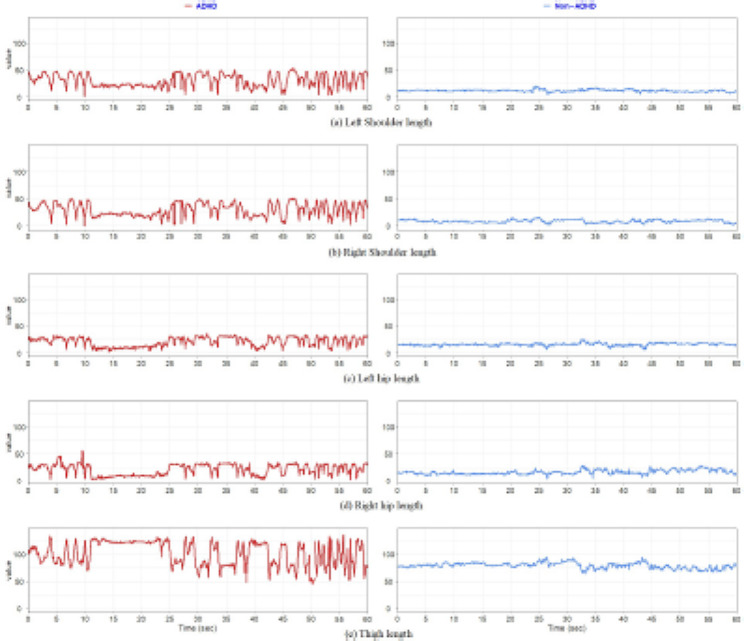
Curve plots of five length-related skeleton parameters between one patient with ADHD and one patient without ADHD.

**Fig. 5:**
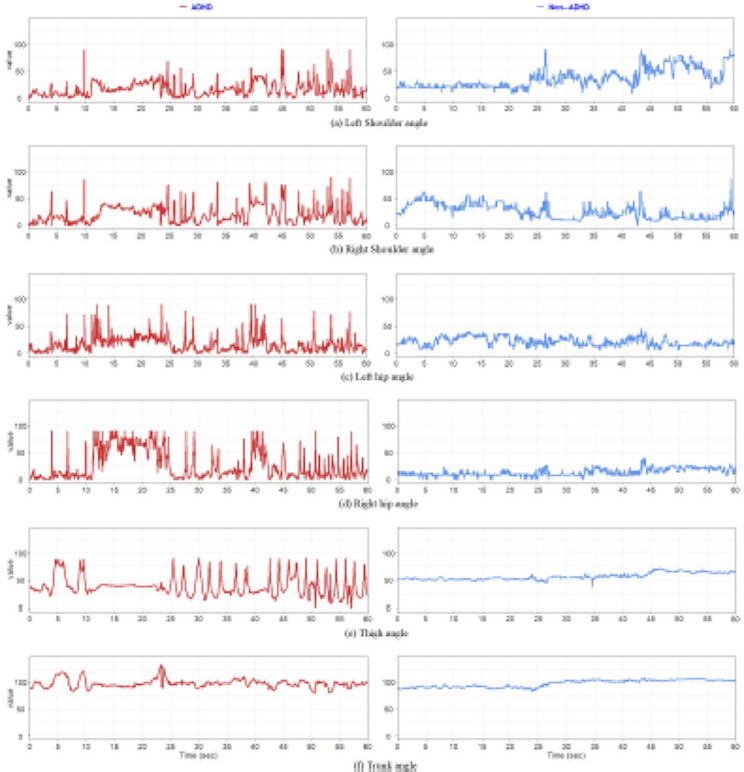
Curve plots of six angle-related skeleton parameter time series between one patient with ADHD and one patient without ADHD

To explore and compare detected movement data between the ADHD and non-ADHD groups visually, the curves of the five length-related and six angle-related skeleton parameter time series between one patient with ADHD (red curves) and one patient without ADHD (blue curve) were plotted and are presented in Figs. 4 and 5, respectively. Curves corresponding to the same patient were plotted for a length of 60 s only because of visual clarity. The curves of the patient with ADHD fluctuated more and were larger than those of the patient without ADHD. This finding indicates that the patient with ADHD exhibited frequent and larger movements of the corresponding body part, especially the shoulder, hip, and thigh. We used the *t* test to compare the data of each single feature descriptor of the skeleton parameter’s averaged variance between the groups. The results are listed in Table 2. Compared with the non-ADHD group, the ADHD group had larger means in all cases of single feature descriptors and larger variances in eight cases. Each single feature descriptor significantly differed between the ADHD and non-ADHD groups. Because a larger averaged variance indicated more and larger fluctuations in a skeleton parameter’s time series, the result of the statistical comparison was verified using the visual observation findings.

**Table 2:** Statistical comparison of 11 single feature descriptors between ADHD and non-ADHD groups

Feature Descriptor	ADHD	Non-ADHD	*p*-value
Left shoulder angle	123.39 ± 17.35	74.36 ± 21.33	0.0008***
Right shoulder angle	141.33 ± 19.57	73.57 ± 21.37	< 0.0001***
Left hip angle	99.21 ± 14.22	51.62 ± 12.00	< 0.0001***
Right hip angle	127.43 ± 18.27	75.74 ± 19.27	0.0002***
Thigh angle	157.89 ± 32.81	15.37 ± 6.62	< 0.0001***
Trunk angle	50.60 ± 17.65	8.60 ± 2.53	< 0.0001***
Left shoulder length	39.69 ± 7.11	10.15 ± 1.70	< 0.0001***
Right shoulder length	39.78 ± 7.22	9.77 ± 1.66	< 0.0001***
Left hip length	16.72 ± 2.91	5.49 ± 1.14	< 0.0001***
Right hip length	18.29 ± 3.13	5.93 ± 1.09	< 0.0001***
Thigh length	164.99 ± 41.86	22.51 ± 7.31	< 0.0001***

Angle: degree/frame, length: pixel/frame

* *p* < 0.05, ** *p* < 0.01, *** *p* < 0.001

**Fig. 6:**
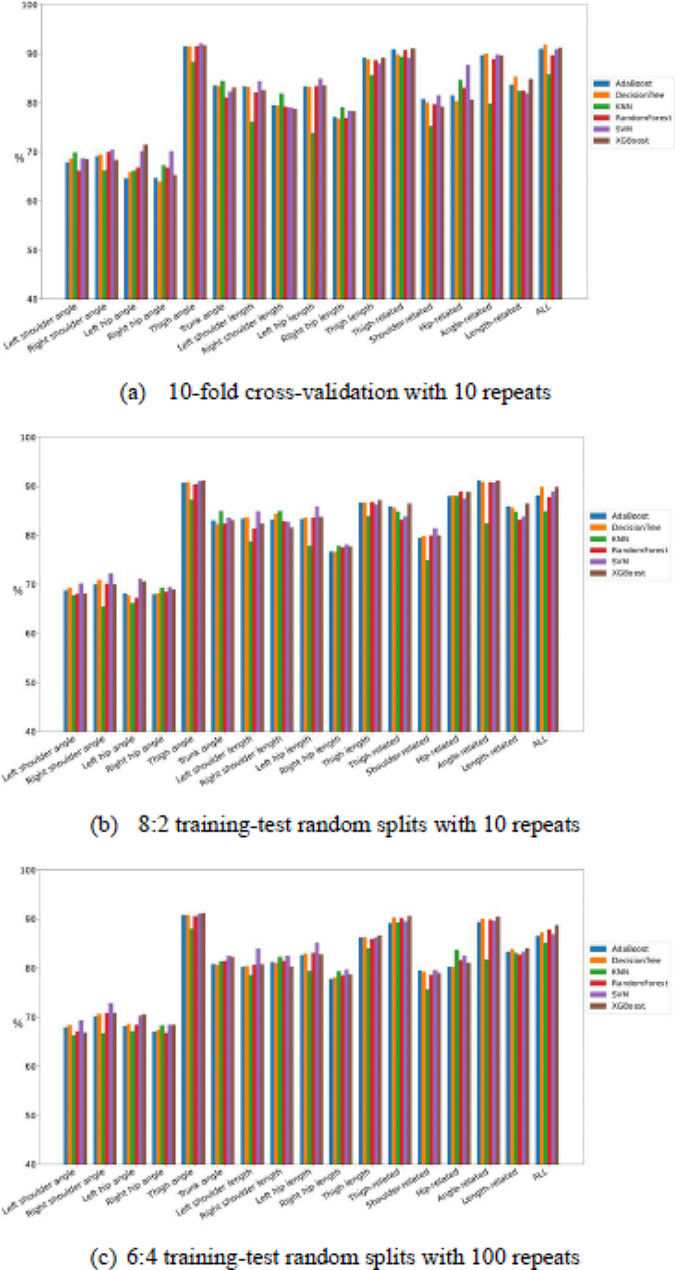
Comparison of the classification test performance of accuracy between classifiers among all feature combinations

**Fig. 7:**
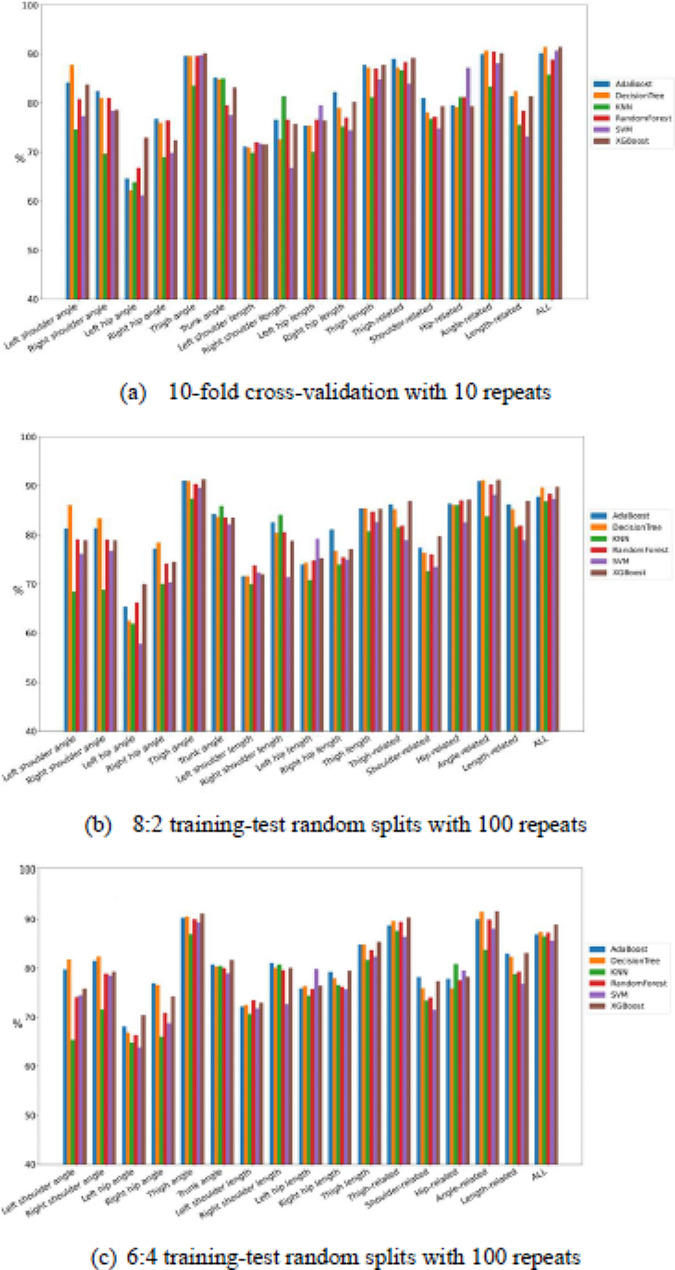
Comparison of the classification test performance of sensitivity between classifiers among all feature combinations

**Fig. 8:**
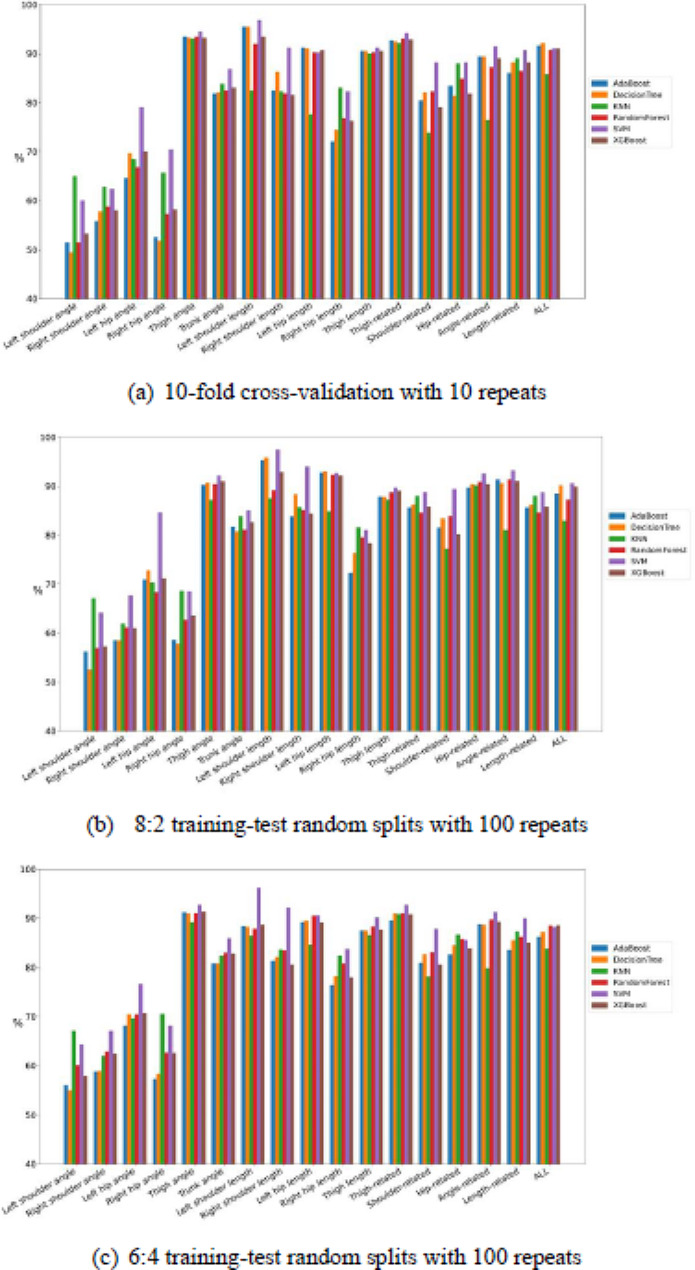
Comparison of the classification test performance of specificity between classifiers among all feature combinations

To determine the discriminability of each single feature descriptor between the ADHD and non-ADHD groups, we determined the cutoff, and the results are presented in Table 3. The feature descriptor “thigh angle” achieved the most favorable result with an optimal cutoff of 42.39, an accuracy of 91.03%, a sensitivity of 90.25%, a specificity of 91.86%, and an AUC of 94.00%. The second-best feature descriptor was “thigh length,” which yielded an accuracy of 86.21%, a sensitivity of 84.28%, a specificity of 88.08%, and an AUC of 93.04%, and the corresponding optimal cutoff was 45.57.

**Table 3:** Cutoff analysis of 11 single feature descriptors between ADHD and non-ADHD groups

Feature descriptor	Optimal cutpoint	Accuracy	Sensitivity	Specificity	AUC
Left shoulder angle	64.91	68.97	80.70	57.57	78.05
Right shoulder angle	75.94	73.20	79.65	66.78	82.25
Left hip angle	65.79	68.91	67.17	70.91	79.34
Right hip angle	69.49	68.77	78.02	59.20	74.69
Thigh angle	42.39	91.03	90.25	91.86	94.00
Trunk angle	15.41	82.77	82.41	83.10	92.89
Left shoulder length	20.52	81.34	72.76	90.00	92.34
Right shoulder length	18.13	79.25	77.43	81.02	93.20
Left hip length	9.71	82.09	75.84	88.31	90.59
Right hip length	9.18	77.94	80.19	75.43	91.02
Thigh length	45.57	86.21	84.28	88.08	93.04

**Fig. 9:**
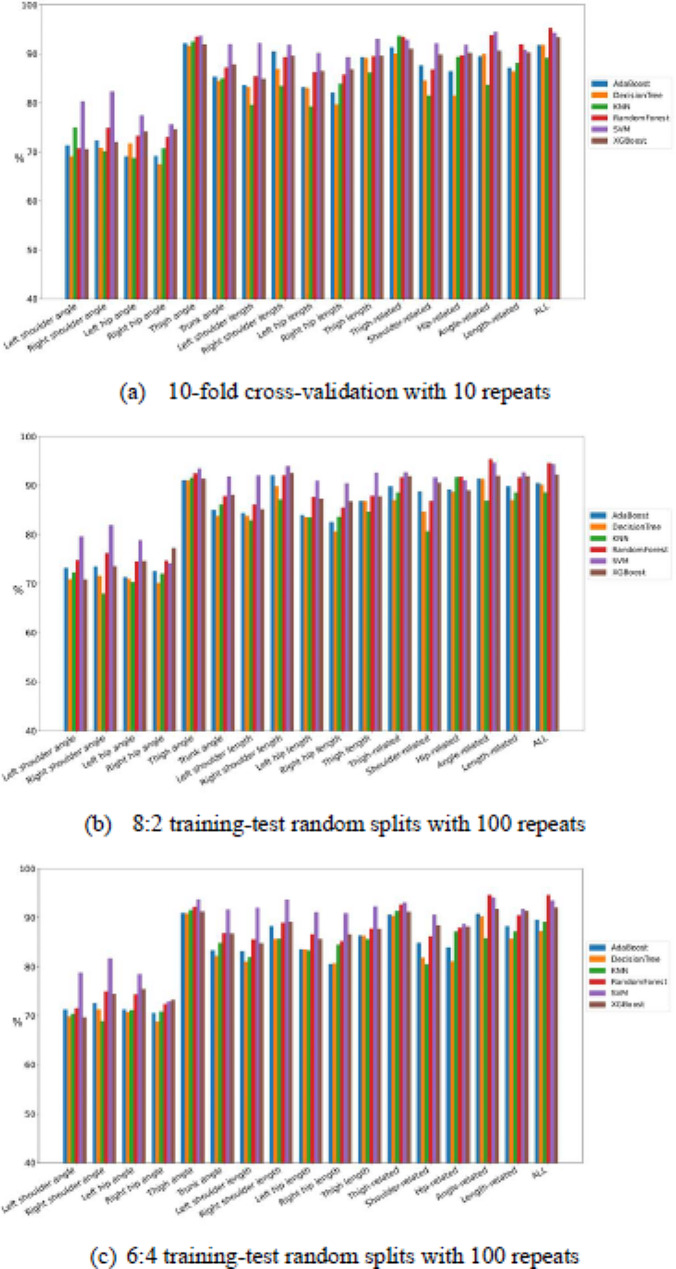
Comparison of the classification test performance of AUC between classifiers among all feature combinations

Figures 6, 7, 8 and 9 present the comparisons of sensitivity, specificity, accuracy, and AUC by three resampling strategies among six classifiers for each of the 17 feature sets. Some classifiers exhibited satisfactory classification performance for all four indices for each of the four feature sets: thigh angle, thigh related, angle related, and all. By the 10-fold cross-validation with 10 repeats, all classifiers for the thigh angle feature set achieved values of over 85% for all four indices, except KNN. Among all “feature set + classifier” combinations, “All + decision tree” exhibited the highest sensitivity (91.40%), “left shoulder length + SVM” exhibited the highest specificity (96.80%), “thigh angle + SVM” exhibited the highest accuracy (92.10%), and “All + Random Forest” exhibited the highest AUC (95.22%). By the 8:2 training-test random splits with 100 repeats, all classifiers for the thigh angle feature set achieved values of over 87% for all four indices, except KNN. Among all “feature set + classifier” combinations, “thigh angle + XGBoost” exhibited the highest sensitivity (91.30%), “left shoulder length + SVM” exhibited the highest specificity (97.40%), “thigh angle + XGBoost” exhibited the highest accuracy (91.10%), and “angle-related + Random Forest” exhibited the highest AUC (95.38%). By the 6:4 training-test random splits with 100 repeats, all classifiers for the thigh angle feature set achieved values of over 86% for all four indices, except KNN. Among all “feature set + classifier” combinations, “angle-related + XGBoost” exhibited the highest sensitivity (91.60%), “left shoulder length + SVM” exhibited the highest specificity (96.25%), “thigh angle + XGBoost” exhibited the highest accuracy (91.20%), and “angle-related + Random Forest” exhibited the highest AUC (94.53%). Tables 4, 5 and 6 present the averaged rankings of all feature combinations corresponding to each classification performance index with three resampling strategies. By the 10-fold cross-validation with 10 repeats, the “thigh angle” feature set ranked first in terms of its specificity and AUC, second in terms of its accuracy, and third in terms of its sensitivity. The “All” feature combination ranked first in terms of its accuracy and sensitivity, second in terms of its AUC, and fourth in terms of its specificity. The “thigh-related” feature combination ranked second in terms of its specificity, third in terms of its accuracy and AUC, and fourth in terms of its sensitivity. By the 8:2 training-test random splits with 100 repeats, the “thigh angle” feature set ranked first in terms of its accuracy and sensitivity, and fourth in terms of its specificity and AUC. The “angle-related” feature combination ranked first in terms of its AUC, second in terms of its accuracy and sensitivity, and fourth in terms of its specificity. The “All” feature combination ranked second in terms of its AUC, third in terms of its sensitivity and AUC, and fourth in terms of its accuracy. By the 6:4 training-test random splits with 100 repeats, the “thigh angle” feature set ranked first in terms of all four indices. The “angle-related” feature combination ranked second in terms of its accuracy, sensitivity, and AUC. The “All” feature combination ranked third in terms of its accuracy, sensitivity, and AUC.

**Table 4:** Averaged ranking of all feature combinations corresponding to each classification performance index by the 10-fold cross-validation with 10 repeats

Features	Accuracy average rank	Sensitivity average rank	Specificity average rank	AUC average rank
Left shoulder angle	15.66	7.83	16.83	15.50
Right shoulder angle	14.83	9.83	15.66	14.66
Left hip angle	15.66	16.66	14.00	15.66
Right hip angle	15.83	14.66	15.50	15.83
Thigh angle	**2.16	***2.66	*1.66	*1.33
Trunk angle	8.83	6.83	9.83	7.83
Left shoulder length	10.83	15.50	**2.66	8.83
Right shoulder length	7.50	13.00	9.50	11.33
Left hip length	11.83	12.50	6.66	8.16
Right hip length	11.66	10.66	12.16	12.5
Thigh length	6.16	5.33	5.16	4.83
Thigh-related	***3.16	4.00	**2.66	***2.83
Shoulder-related	8.33	10.66	11.33	11.16
Hip-related	7.50	8.16	9.16	8.00
Angle-related	4.16	**2.50	7.33	4.50
Length-related	6.83	9.83	7.33	7.16
All	*1.83	*1.50	4.83	**2.16

Bold underline: rank first

* rank first

** rank second

*** rank third

**Table 5:** Averaged ranking of all feature combinations corresponding to each classification performance index by the 8:2 training-test random splits with 100 repeats

Features	Accuracy average rank	Sensitivity average rank	Specificity average rank	AUC average rank
Left shoulder angle	15.67	10.50	16.83	15.33
Right shoulder angle	14.67	11.17	16.00	14.83
Left hip angle	15.83	17.00	13.83	16.00
Right hip angle	15.83	14.17	15.17	15.83
Thigh angle	*1.83	*1.17	4.50	3.33
Trunk angle	9.00	6.83	11.17	9.33
Left shoulder length	9.17	15.33	*2.17	11.00
Right shoulder length	8.83	10.17	7.33	***3.17
Left hip length	8.33	12.83	**3.17	11.33
Right hip length	12.83	12.17	12.83	12.33
Thigh length	5.50	6.33	6.83	8.50
Thigh-related	6.83	6.83	8.50	5.50
Shoulder-related	12.17	12.00	11.33	9.67
Hip-related	***3.33	4.00	***4.00	6.17
Angle-related	**2.50	**2.50	4.50	*2.50
Length-related	7.17	7.17	7.83	5.50
All	3.50	***2.83	7.00	**2.67

Bold underline: rank first

* rank first

** rank second

*** rank third

**Table 6:** Averaged ranking of all feature combinations corresponding to each classification performance index by the 6:4 training-test random splits with 100 repeats

Features	Accuracy average rank	Sensitivity average rank	Specificity average rank	AUC average rank
Left shoulder angle	16.33	12.17	16.83	16.00
Right shoulder angle	14.33	9.00	15.67	14.67
Left hip angle	15.00	17.00	14.17	15.00
Right hip angle	16.33	14.67	15.33	16.33
Thigh angle	*1.17	*1.50	*1.50	*2.17
Trunk angle	9.33	7.83	11.17	9.50
Left shoulder length	10.17	15.17	4.83	11.33
Right shoulder length	8.83	8.83	9.33	5.67
Left hip length	7.17	11.67	***4.33	10.17
Right hip length	12.50	10.50	12.67	11.83
Thigh length	4.83	5.00	6.17	7.17
Thigh-related	2.17	2.67	**1.83	3.33
Shoulder-related	12.33	13.17	11.00	10.17
Hip-related	8.67	10.33	8.67	8.83
Angle-related	**3.50	**2.00	5.33	**2.50
Length-related	6.50	7.67	7.17	5.33
All	***3.83	***3.83	7.00	***3.00

Bold underline: rank first

* rank first

** rank second

*** rank third

## Discussion

This study revealed that variances in our measurements were significantly higher in the ADHD group than in the non-ADHD group. The classification performance of our proposed model was excellent, with sensitivity, specificity, accuracy, and AUC of 91.40%, 96.80%, 92.10%, and 95.22%, respectively. The main reason was defined feature descriptors, namely variances of skeleton parameters extracted from the detected subject’s skeleton, were highly discriminable between ADHD and non-ADHD groups, resulting in well-trained classification models and the correspoding superior generalization capability. Thus, variances in measurements may be useful and objective markers that can assist in ADHD diagnosis.

The SNAP-IV questionnaire was initially proposed to assess ADHD symptoms in accordance with *DSM*, *Third Edition* [23, 24]. Although the SNAP-IV score has high validity and reliability [25–27], a study reported poor interrater agreement between parents and teachers [28]. In addition, the parents’ scorings of inattention and hyperactivity/impulsivity are favorable predictors for diagnosis in research but not in clinical diagnosis, whereas the teachers’ scorings of only hyperactivity/impulsivity are satisfactory predictors for diagnosis both in research and clinical settings [26]. These discrepancies between parents’ and teachers’ scorings may lead to diagnostic uncertainty. In this study, we used skeleton detection to objectively evaluate the activities of the patients with ADHD. We observed that the activities of the patients with ADHD were significantly more than those of the patients without ADHD, indicating higher variances in our measurements.

Nowadays, there are some movement detection methods available to assist in diagnosing ADHD, including accelerometers, actigraphy, infrared, and ultra-wideband radar. Each method has its strengths and weaknesses. Accelerometers and actigraphy are mostly worn on the wrist or ankle for detecting specific movements of subjects. Both sensors can be used at home or school instead of a laboratory [9]. However, they need to be attached to the subject’s body, limiting their ecological validity. In addition, only body parts equipped with sensors can be recorded. The difference between accelerometers and actigraphy is that accelerometer analyzes the subject’s movements during normal daily activities and the recording time is limited by the power of battery [29], whereas actigraphy studies the subject’s sleep efficiency and the recording is limited by low sampling rate [30]. Regarding to infrared, the strength of infrared is noncontact without placing any type of sensor in the body of the subjects [10]. However, infrared detection is easily interfered by light or other noise. In addition, it usually requires the use of special detection and software equipment. For ultra-wideband radar, it is also a noncontact method without any sensor attached to the subject’s body. Moreover, it can be applied in various situations, such as during a test or in a naturalistic setting [11]. The disadvantages of ultra-wideband radar are that it needs to be used in a limited space and the surrounding moving objects of the environment will affect radar detection. Our proposed method also provides a noncontact method and can use the video for analysis from a regular camera. This method has good classification results between ADHD and non-ADHD in a short detection time. In addition, the detection can be conducted during regular consultation and will not affect normal visiting behavior. The weaknesses of our method are two folds: (1) the detection data may be interfered by human body occlusion. (2) the method needs to be used in a limited space (Table 7). However, in our consulting room, these two shortcomings can be overcome through experimental design. Furthermore, we also compare the performance metrics of our proposed method and other diagnostic methods that are using video recording (Table 8). Although the studies from Li et al. demonstrated high precision, they used adults as the study subjects and the case number was limited to 17 [31, 32]. Sempere-Tortosa et al. used Microsoft Kinect V.2. to track joint movements of human bodies in children with ADHD and controls. Although their results showed that the differences in movement were significant for 14 of the 17 joints between two groups, this method requires special detection and software equipment [33]. Our proposed method using only a regular camera is a convenient way to differentiate ADHD children and controls with high performance indexes by selecting only one joint feature.

**Table 7:** Comparison the strengths and weaknesses of different methods in evaluation the movement abnormalities in patients with ADHD

Devices	Strengths	Weaknesses
Accelerometers	1. Subjects can use the devices at home or school instead of a laboratory.	1. The sensors need to be attached to the subject’s body, limiting their ecological validity. 2. Only body parts equipped with sensors are recorded. 3. Recording time is limited by the power of battery.
Actigraphy	1. Subjects can use the devices at home or school instead of a laboratory.	1. Actigraphy is mostly used to record sleep activities. 2. Only body parts equipped with sensors are recorded. 3. Recording is limited by low sampling rate.
Infrared	1. Can track and record joint movements without placing any type of sensor in the body of the subject.	1. Infrared detection is easily interfered by light or other noise. 2. It usually requires the use of special detection and software equipment.
Ultra-wideband radar	1. It is a noncontact method without any sensor attached to the subject’s body. 2. It can be applied in various situations, such as during a test or in a naturalistic setting.	1. Need to be used in a limited space. 2. The surrounding moving objects of the environment will affect radar detection.
Our method (OpenPose)	1. It is a noncontact method. 2. Can use the video for analysis from a regular camera. 3. The method yields good classification results in a short detection time during regular consultation will not affect normal visiting behavior.	1. The detection data may be interfered by human body occlusion. 2. Need to be used in a limited space.

**Table 8:** Comparison the performance metrics between different methods using video in diagnosis of ADHD

Authors	Patient number	Methods	Performance metrics
Li et al. [31]	7 adult ADHD, and 10 controls	Videos are recorded in a series of consecutive tests for 70–80 min and subjects’ motions are analyzed by action-based analysis.	Using Pose C 3D network Precision: 100% F1: 88.9% Accuracy: 88.2% AUC: 83.0%
Li et al. [32]	7 adult ADHD, and 10 controls	Videos are recorded in four continuous dialogue tasks for 70–80 min and subjects’ motions are analyzed by time-action based analysis.	Using 3D-CNN structure network Sensitivity: 100% Precision: 90.9% Accuracy: 94.1% AUC: 97.0%
Sempere-Tortosa et al. [33]	32 ADHD children with mean age 9y9m, and 33 controls with mean age 9 y 8 m	Track the joint movements of human bodies by Microsoft Kinect V.2. during a workshop in the classroom.	The differences found between ADHD and control groups are significant for 14 of the 17 joints, especially in spine base, left wrist, right elbow, right wrist, and right hip, with oscillating between 0.83 and 1.12.
Our method	48 ADHD children with mean age 7y6m, and 48 controls with mean age 7 y 8 m	Videos are recorded during regular consultation for 4–6 min and subjects’ motions are detected by OpenPose and averaged variances of skeleton parameters are analyzed.	Thigh angle used as a single feature descriptor Accuracy: 91.03% Sensitivity: 90.25% Specificity: 91.86% AUC: 94.00%

Pose C3D: 3D-CNN structure

OpenPose is used to localize anatomical key points or regions; it focuses on identifying the body parts of individuals. Few studies have used a noncontact method, such as Kinect, to record the number of movements of patients with ADHD, and studies have reported significant differences in the extent of objective movement between patients with ADHD and controls [33, 34]. Our study is the first to use OpenPose to objectively analyze the body movements of patients with and without ADHD in a consulting room. Classification performance was satisfactory, with the AUC being as high as 95.22%. Our proposed method can thus be an objective and reliable tool that can assist in ADHD diagnosis.

In this study, thigh angle and length had the highest discriminating power between the ADHD and non-ADHD groups. Because the chair in the consulting room can be rotated by patients, body spin was the dominant movement. Sempere-Tortosa et al. investigated the movement patterns of patients with ADHD at a school by using the Kinect device, which can measure the movements of different body parts. They determined that turning of the head when children with ADHD change their attentional focus to a different stimulus is the most common movement pattern [34]. Another study used two triaxial accelerometers as sensors that were applied to the wrist and ankle of the dominant arm and leg to record the movements of patients with ADHD and controls 24 h a day. They believed that the hands and legs are the most active body parts of patients with ADHD [35]. Gross used a swivel chair to examine patients with ADHD. He determined that most patients attempted to spin the chair in one direction in the consulting room [36]. Consistently, our previous study indicated that the most frequent movement in patients with ADHD tended to be hand-tapping at school, as revealed by smart watch recordings [37]. Thus, the predominant movement pattern may differ depending on the environment. In a consulting room with a rotatable chair, as was the case in the current study, body spin calculated using the thigh angle feature may be a sensitive tool to differentiate between patients with and without ADHD.

This study has several limitations. First, sample sizes for each ADHD subtype, especially ADHD-I, were small. Thus, the results may not be generalizable to all ADHD subtypes. Studies should enroll more patients with different ADHD subtypes to comprehensively evaluate the diagnostic value of the objective tool in all the three subtypes. Second, uncontrollable factors may affect children’s activities in a consulting room, including food intake on the day of consulting and examinations, sleep quality before examination, and other emotion problems. Studies should include a questionnaire to determine the relationship between these corresponding factors and children’s activities. Third, although we had excluded children with a history of psychotic disorders from the ADHD group and included patients with headache, epilepsy, and dizziness only in non-ADHD group, underdiagnosis of autistic spectrum disorders or other movement disorders with comorbidity in both groups may still happen and interfere with our analytic results.

## Conclusions

Most patients with ADHD have ADHD-H or ADHD-C subtypes and exhibit the main symptom of hyperactivity. In this study, the proposed approach based on movement quantization through the analysis of variance in patients’ skeletons detected in outpatient videos effectively differentiated between patients with and without ADHD. The experimental results revealed that compared with the non-ADHD group, the ADHD group had significantly larger means in all the cases of single feature descriptors. Thigh-related feature descriptors played a key role in distinguishing the movements of patients between the ADHD and non-ADHD groups. In conclusion, the proposed machine learning–based approach can serve as a reliable model for evaluating and classifying patients into ADHD and non-ADHD groups objectively and automatically and can help physicians make clinical decisions regarding ADHD diagnosis.

## Data Availability

No datasets were generated or analysed during the current study.

## Abbreviations

ADHD: Attention-deficit/hyperactivity disorder
DSM: Diagnostic and statistical manual of mental disorders
OA: Osteoarthritis
KAM: knee adduction moment
SNAP-IV: Swanson, Nolan, and Pelham Rating Scale IV
AUC: Receiver operating characteristic curve
AdaBoost: Adaptive boosting
CART: Classification and regression tree
KNN: k-Nearest neighbors
SVM: Support vector machine
XGBoost: Extreme gradient boosting
